# Quality of life among young people in Norway during the COVID-19 pandemic. A longitudinal study

**DOI:** 10.1007/s00787-022-02023-5

**Published:** 2022-06-22

**Authors:** Stine Lehmann, Ellen Haug, Ragnhild Bjørknes, Gro Mjeldheim Sandal, Lars T. Fadnes, Jens Christoffer Skogen

**Affiliations:** 1 Department of Health Promotion and Development, Faculty of Psychology, The University of Bergen, Postboks 7807, 5020 Bergen, Norway; 2 NLA University College, Bergen, Norway; 3 The Norwegian Center for Child Behavioral Development, Oslo, Norway; 4 Department of Psychosocial Science, Faculty of Psychology, The University of Bergen Norway, Bergen, Norway; 5 Department of Global Public Health and Primary Care, Faculty of Medicine, University of Bergen, Bergen, Norway; 6 Department of Addiction Medicine, Bergen Addiction Research, Haukeland University Hospital, Bergen, Norway; 7 Department of Health Promotion, Norwegian Institute of Public Health, Bergen, Norway; 8 Alcohol and Drug Research Western Norway, Stavanger University Hospital, Stavanger, Norway; 9 Centre for Evaluation of Public Health Measures, Norwegian Institute of Public Health, Oslo, Norway

**Keywords:** COVID-19, Youth, Quality of life, Parental stress, Longitudinal study

## Abstract

In this study, we aimed to examine health-related quality of life during the COVID-19 pandemic among a general sample of young people in Norway aged 11–19 years. More specifically, we examine: (1) Change over 2 time-points in five health-related quality of life dimensions, (2) Whether sociodemographic- and COVID-19-related factors contributed to change in these five dimensions, (3) Whether parental stress and socioeconomic status at T1 interacted with change in health-related quality of life across T1 and T2. Data collection lasted from April 27th to May 11th, 2020 (T1), and from December 16th, 2020, to January 10th, 2021 (T2). Youth aged 11–19 years (*N* = 2997) completed the KIDSCREEN-27, COVID-19 related and sociodemographic items. Parents (*N* = 744) of youth aged 15 years and younger completed the parental stress scale and sociodemographic items. Physical and psychological wellbeing declined significantly from March to December 2020. Subscale scores for social support and peers increased. Controlling for a broad number of sociodemographic and COVID-19-related factors did not make an overall impact on the estimates. Those worried about infection, older aged, girls, and youth born outside Norway had a steeper decline in health-related quality of life subdimensions from T1 to T2. In the wake of the COVID-19 pandemic, we warrant special attention to the recovery of youth's physical and psychological wellbeing.

**Supplementary Information:**

The online version contains supplementary material available at 10.1007/s00787-022-02023-5.

## Introduction

The COVID-19 pandemic caused a massive worldwide effort to suppress the highly contagious and potentially deadly virus [1]. Young people have been less vulnerable to severe symptoms from the SARS-CoV-2 virus infection [2]. However, extensive social distancing measures caused significant changes in young people’s daily lives with evidence of impact on their mental health and health behaviors [3–10]. In the wake of the disease-suppressive measures implemented, the current study examines how the pandemic has impacted young people’s quality of life (QoL).

World Health Organization (WHO) defines QoL as individuals’ perception of their position in life, within the context of the culture, value systems, their goals, expectations, standards, and concerns [11]. The term health-related QoL (HRQoL) is a multifactorial construct [12], with physical health, psychological state, and social relationships being among the main components [11, 13, 14].

According to UNCRC (29.1), children and youth have a right to the “development of personality, talents and mental and physical ability to their fullest potential” [15]. HRQoL is a resource for adaptation and healthy growth for young people, and if reduced, young people are less likely to cope effectively, develop normally, and mature into healthy adults [16]. Thus, HRQoL has been suggested as an universal outcome to be evaluated in large-scale studies of the impact of policies or interventions [15].

A German study of 7–17 year old’s, found lower levels HRQoL in June 2020 compared to pre-pandemic scores [17], with even further decline in January 2021 [18]. However, a recent review yielded inconclusive results as three of six identified studies concluded with reduced HRQoL during the pandemic, whereas the other studies did not find a significant decline [19].

In Norway, mean scores below European norms on KIDSCREEN-10 have been reported for children [20] and youth [21] during the first weeks of the lockdown. Boys and those with parents with higher education generally had better HRQoL, whereas confirmed or suspected COVID-19 and having been isolated or quarantined were associated with lower HRQoL. Surprisingly, social inequality in life satisfaction has been smaller during the restrictions than in pre-pandemic periods in Norway [22]. This is in contrast to findings from Germany, where youths with low socioeconomic status, migration background and limited living space were more negatively affected [17].

There is evidence that COVID-19 impacted many families’ wellbeing and led to increased parental stress [23, 24]. The social distancing measures have made families spend considerably more time at home, and parents have had increased responsibility for educational follow-up due to online or home-schooling. The importance of parental stress for child wellbeing has been demonstrated, where a reduction in parental stress during the pandemic yield increased wellbeing among the children [25]. Further, connectedness to caregivers before the pandemic has predicted wellbeing among children during the outbreak [26]. Other family factors such as single-parent households and difficult work situation have been associated with worse mental and social health among children during this period [10]. A Norwegian study showed that single-parent households reported lower scores on maternal wellbeing when controlling for mothers’ income, which was related to low HRQoL scores among the children [20]. How young people might adjust to increased parental stress over time, and whether parent-related factors impact the youth HRQoL during the pandemic is not known.

To sum up, HRQoL is crucial for adaptation and healthy development, and substantial concerns have been raised regarding the impact of extensive social distancing measures on young people. Still, knowledge on stability and change in HRQoL among young people during the pandemic is scarce. Further, previous studies on young people’s HRQoL during the COVID-19 pandemic have utilized short-form overall indexes of HRQoL. However, as HRQoL is a multifactorial construct [11, 13, 14], the COVID-19 context might affect the domains differently. To the best of our knowledge, no study has addressed the possible differential impact of COVID 19 on the diverse dimensions that constitute HRQoL. There is also a lack of studies following young people over an extended period during the pandemic. Further, a lack of longitudinal designs and that the research only included data from either parents or young people only are among the limitations in many of the studies on mental health among young people during the COVID-19 [27]. We aim to fill these knowledge gaps by employing data from parents and youth responses during the lockdown and again nine months later. Using a multifactorial measure of HRQoL, we examine five different HRQoL domains among young people during the pandemic.

We use data from a longitudinal study to examine HRQoL among young people aged 11 to 19 years during the Covid-19 pandemic. More specifically, we examine:

1. Change over 2 time-points in five dimensions of youth HRQoL (physical wellbeing; psychological wellbeing; parent relations and autonomy; peers and social support; and school environment).
2. Whether sociodemographic- and COVID-19-related factors contributed to change in the five dimensions of HRQoL.
3. Whether parental stress and socioeconomic status (SES) at the start of the pandemic period interacted with change in HRQoL across time.

## Methods

### Procedure and study sample

Data were obtained from the COVID-19 Young study, a longitudinal study of youth aged 11–19 years attending secondary and high schools within Bergen, Norway [8]. The first data collection (T1) started on April 27th, 2020, during the seventh week of the national lockdown, and closed on May 11th. The second data collection (T2) began on December 16th, 2020, and ended on January 10th 2021. During T1 the weekly infection rates were close to zero, with 0–4 /100 000 in the county of Vestland. During T2, the numbers were also low, with weekly cases of 22/ 100 000 [28]. Local restrictions in the second data collection implied partly closed schools, and sports- and leisure activities were put on hold. At the lower secondary level and upper secondary level, a larger proportion had digital home-schooling part of the time compared to younger age groups [29].

We recruited two subsamples. Cohort 1 consisted of young people aged 11–15 years whose parents participated in the population-based study Bergen in change (BiE-study) [30]. A random selection of 81,170 individuals from a total of 224,000 adult inhabitants in Bergen, Norway, were drawn from the National Population Registry and invited to participate. We distributed invitations by email and SMS. Of the 29,535 (36% response rate) participants, 1565 reported having children below 16 years in their household and were thus eligible to answer the Parental Stress Scale (PSS) for this sub-study. Parents completed PSS a secure online platform. Scores were assigned to individual cases defined by participating youth in the data file.

Upon parental consent to their children participating and providing contact information, we invited the youth aged 11–15 years to participate in the present study. A total of 1565 youth was contacted in cohort 1 at time-point 1. Consenting parents were more often women (Cramérs V: 0.069, *P* < 0.001), older (Cramérs V: 0.092, *P* < 0.001), had higher educational attainment (Cramérs V: 0.155, *P* < 0.001) and household income (Cohen’s D: 0.19, *P* < 0.001) and had less often shared residence for the child (Cramérs V: – 0.054, *P* = 0.006) when compared to non-consenting parents. These differences were very small to small [8].

Cohort 2 were young people aged 16–19 years, attending high school. For this cohort, the county of Vestland provided phone numbers from their schools’ contact registers. A total of 5,947 youth in cohort 2 were contacted at time-point 1. For this age group, we could not assign PSS scores of parents, as this cohort was not recruited through their parents.

The invitation procedures were the same for cohorts 1 and 2 at both time points. We recruited youth via SMS with a link to a secure online platform containing an information letter and a—survey estimated to take 15 to 30 min to answer. Two SMS reminders were sent. Participants were included in a lottery for a new cellphone at both time points.

In wave 1, a total of 7512 youth was invited to participate. Of these, 843 (54%) in cohort 1 and 2154 (36%) in cohort 2 responded, yielding a total of 2997 (40% response rate) youths completing the T1 survey. Among those, 61 participants did not report their age and gender, and 276 participants had no valid KIDSCREEN subscales and were excluded from further analyses. All participants from wave 1 were invited to answer the survey on T2. A total of 1,493 (58%) of the baseline analytical sample also completed the second survey. Those participating at both time points were more likely to be girls and younger than those who only responded at baseline. They were more likely to live with both parents and less likely to report that one of their parents was laid off (at baseline). They also scored slightly higher on the school-related subscale. When comparing the two groups, no other differences were observed (details described in Supplementary Information, Table 1).

**Table 1:** Summary statistics of main variables

Variables	Boys, *N* = 1085^a^	Girls, *N* = 1575^a^	*p*-value^b^	Effect size
Age groups (valid *N* = 2660)			0.072	51.6% (0.035)^c^
12–15 years	306 (28%)	395 (25%)		
16–19 years	779 (72%)	1,180 (75%)		
Country of birth (valid *N* = 2660)			0.5	0.013^d^
Norway	1013 (93%)	1460 (93%)		
Other country	72 (6.6%)	115 (7.3%)		
Living arrangements (valid *N* = 2660)			0.033	0.051^d^
Both parents	914 (84%)	1268 (81%)		
Mother or father	151 (14%)	262 (17%)		
Other	20 (1.8%)	45 (2.9%)		
Parent laid off (% yes; valid *N* = 1898)	173 (23%)	241 (21%)	0.4	0.021^d^
Worry I will get infected (valid *N* = 2646)			< 0.001	56.8% (0.132)^c^
Not true	663 (61%)	765 (49%)		
Somewhat true	367 (34%)	668 (43%)		
Completely true	49 (4.5%)	134 (8.6%)		
Worry, family infected (valid *N* = 2647)			< 0.001	59.2% (0.177) ^3^
Not true	172 (16%)	124 (7.9%)		
Somewhat true	440 (41%)	506 (32%)		
Completely true	467 (43%)	938 (60%)		
Worry about future (valid *N* = 2644)			< 0.001	55.7% (0.106) ^c^
Not true	568 (53%)	664 (42%)		
Somewhat true	341 (32%)	564 (36%)		
Completely true	169 (16%)	338 (22%)		
Physical wellbeing (valid *N* = 2584)^e^	44 (9)	41 (9)	< 0.001	0.380^f^
Psychological wellbeing (valid *N* = 2574)^e^	45 (9)	41 (8)	< 0.001	0.578^f^
Autonomy and parent relation (valid N = 2550)^e^	52 (9)	50 (9)	< 0.001	0.243^f^
Social support and Peers (valid *N* = 2560)^e^	46 (9)	44 (9)	< 0.001	0.183^f^
School-related (valid *N* = 2555)^e^	43 (9)	41 (9)	< 0.001	0.215^f^

^a^Statistics presented: *n* (%); mean (SD)

^b^Statistical tests: chi-square test of independence for categorical data; two-sample *t*-test for continuous data

^c^Probability that variable for girls is larger than variable for boys, Cramer’s V in parenthesis

^d^Cramer’s V

^e^*T*-score at baseline

^f^Cohen’s D

At baseline, valid parental-reported parental stressors and rewards (PSS) were available for 744 out of 748 (99.5%) participants in cohort 1.

### Ethics

Western Norway’s Regional Committee for Medical and Health Research Ethics approved the study (project number 131560). The youth provided informed consent to participate at the start of the survey.

### Measures

Quality of life. At both time points the KIDSCREEN-27 Quality of Life Questionnaire [13], a 27-item self-report of HRQoL during the last week for youth aged 8–18 years was used. KIDSCREEN-27 comprises five dimensions of HRQoL: physical wellbeing (level of physical activity, energy, and fitness, feeling unwell and complaints of poor health); psychological wellbeing (positive emotions and satisfaction with life, absence of loneliness and sadness); parent relations and autonomy (quality of interaction between adolescent and parent, whether s/he feels loved and supported by the family, perceived level of autonomy and financial resources); peers and social support (social relations with friends and peers and perceived support); and school environment (perception of cognitive capacity, learning and concentration, and feelings about school and relationship with teachers). Each item is scored on a five-point Likert scale (1 = “never”/ “not at all” to 5 = “always” or “extremely”). Higher scores indicate higher HRQoL. The reliability, discriminatory power, and validity are good [14, 31].

### Parental stressors and rewards

The Parental Stress Scale (PSS) [32] measures stressful and unsatisfying experiences aspects of being a parent (Parental stressors) and positive and rewarding aspects of being a parent (Parental rewards). Items on parental stressors include for example “I feel overwhelmed by the responsibility of being a parent”. Items on parental rewards include for example “My child (ren) is (are) an important source of affection for me”. Answers are rated on a Likert scale ranging from 1 (“strongly disagree”) to 5 (“strongly agree”). The sum score of the parental stressors subscale ranges from 10 to 50, where a high score signifies a perceived high level of stress in parenthood. The sum score for the parental rewards sub-scale ranges from 8 to 40, where a high score reflects a high level of perceived rewards associated with parenthood. The psychometric properties of the Norwegian version of the PSS have been shown to be satisfactory [33], in line with other research [34]. We dichotomized the PSS subscales based on a median split.

### Covariates measured at baseline

We included the self-reported demographic covariates of age, gender, and country of birth. Age was reported in whole years. In addition, we differentiated between being born in Norway and being born in another country. Additional covariates included the following items: one or both parents temporarily laid off because of the pandemic (yes/no); family structure (single-parent household; both parents; other); worry of contamination self or family member (yes/no); and worries about the future due to the pandemic (yes/no).

### Analyses

First, we computed summary statistics of the sample at baseline across gender (Table 1). For categorical variables, the column-wise proportions were computed along with *p*-values for the chi-square test of independence comparing girls and boys. For the sub-scales of the KIDSCREEN, the mean *T*-scores were computed, and gender differences were assessed using two-sample *t*-tests. Next, change in scores on each of the five subscales of KIDSCREEN were computed in separate linear mixed models using time as the predictor (Table 2). Crude models were first estimated, then models adjusted for the covariates age, gender and country of birth. Finally, fully adjusted models were estimated including the covariates age, gender, country of birth, living arrangement, parent work situation (laid off or not), worries about getting infected, worries about family getting infected, and worries of the future. Thereafter, we tested the potential interaction between time and the covariates on the association with the subscales of KIDSCREEN using mixed linear models (Table 3). For each combination of covariate × KIDSCREEN-subscale we compared the nested model, including the interaction term with a model without the covariate using likelihood ratio tests. For the participants aged 12–15 years, we also investigated the potential interaction between time and parent-reported parental stressors and rewards. The resulting *p*-values from the likelihood ratio tests were presented, and *p*-values < 0.05 were considered indicative of an interaction effect. Finally, for each covariate × KIDSCREEN-subscale combination yielding a significant interaction, we estimated the strata-specific score change from *t*_1_ to *t*_2_ using mixed linear models (Table 4 and Figures). Missing values varied across included variables (from 29% (parent laid off) to 0.5% (worry about family getting infected)), and pairwise deletion was employed to use the maximum number of valid observations available. KIDSCREEN *T*-values were constructed and imputed using IBM SPSS Statistics for Windows, Version 26, according to the KIDSCREEN handbook and the recommended syntaxes from the resource page [13]. For all other data handling and statistical analyses, Stata 17 were used.

**Table 2:** Change in the five dimensions of quality of life (HRQoL) over time

Model	Physical wellbeing	Psychological wellbeing	Autonomy and Parent relation	Social support and Peers	School-related
Unadjusted	− 1.06 (− 0.11)***	− 1.23 (− 0.14)***	− 0.31 (0.03)	0.98 (0.10)***	− 0.04 (< 0.01)
Adjusted for age, gender and country of birth	− 1.06 (− 0.11)***	− 1.25 (− 0.14)***	− 0.35 (0.04)	0.93 (0.10)**	− 0.16 (0.02)
Fully adjusted^a^	− 1.06 (− 0.11)**	− 1.25 (− 0.14)***	− 0.34 (0.04)	0.92 (0.10)**	− 0.14 (0.02)

Absolute score changes in *T*-score and standardized effect sizes. Unadjusted and adjusted for age, gender, and birth country

^a^Adjusted for age, gender, country of birth, living arrangement, parent laid off, worry about getting infected, worry about a family member getting infected and worry about the future

Standardized effect sizes in parentheses based on the following formula: $\frac{{\text{Re} gression\,\,\,coefficient}}{{Polled\;\;s\tan dard\,\,deviation\,\,\left( {t^{1} \;\,and\,\,\,t^{2} } \right)}}$

*P*-values: *< 0.05; ** < 0.01; ***< 0.001

**Table 3:** Test of interaction between time and covariates, *p*-values only

Covariates	Physical wellbeing	Psychological wellbeing	Autonomy and parent relation	Social support and Peers	School-related
Age groups	** < 0.001**	0.391	0.071	0.138	**0.036**
Sex	**0.037**	0.523	0.773	0.798	0.127
Birth country	0.961	0.242	**0.003**	0.472	0.984
Living arrangements	0.634	0.450	0.357	0.105	0.05
Parent laid off	0.894	0.091	0.788	0.569	0.614
Worry I will get infected	**0.042**	0.150	0.147	0.988	0.326
Worry, family infected	0.281	0.588	0.151	0.909	**0.032**
Worry about future	0.440	0.653	0.146	0.973	0.279
Sub-group analyses					
Parental stress scale					
Parental stressors	0.810	0.514	0.502	0.556	**0.012**
Parental rewards	0.476	0.608	0.216	0.260	0.367

Bold font indicates statistically significant estimates (*p* < 0.05)

**Table 4:** Estimated coefficients from stratified analyses of covariate × Kidscreen-subscale combination using mixed linear models

Covariates	Physical wellbeing		Autonomy and parent relation		School-environment	
	Estimate	*p*-value	Estimate	*p*-value	Estimate	*p*-value
Worry I will get infected						
Not true	**− 0.710**	**0.032**	–	–	–	–
Somewhat true	**− 1.162**	**0.003**				
Completely true	**− 3.361**	**0.001**	–	–	–	–
Birth country						
Norway	–	–	− 0.133	0.601	–	–
Other country	–	–	**− 3.371**	**0.001**	–	–
Age groups						
12–15 years	0.370	0.388			**− 0.910**	**0.034**
16–19 years	**− 1.845**	** < 0.001**			0.184	0.531
Worry, family get infected						
Not true	–	–	**1.729**	**0.018**	–	–
Somewhat true	–	–	− 0.423	0.295	–	–
Completely true	–	–	− 0.142	0.675	–	–
Parental stressors						
Below median	–	–	–	–	**− 1.815**	**0.002**
Above median	–	–	–	–	0.285	0.638
Sex						
Boys	− 0.338	0.398	–	–	–	–
Girls	− 1.387	< 0.001	–	–	–	–

Bold font indicates statistically significant estimates

## Results

### Sample characteristics and pandemic experiences

The mean age of the analytical sample (*N* = 2660; cohort 1, *n* = 733; cohort 2, *n* = 1857) was 16 years (SD 1.7), 59% were females, and most participants reported living with both parents (82%) and being born in Norway (93%). Of the participating youth at T1, 22% reported that one or both parents were temporarily laid off due to the pandemic. Worries (somewhat/completely true) of getting infected were reported by 46%, while 89% worried that someone in their family could get infected. Worries that the pandemic may lead to a more difficult future for themselves were reported by 53% of the youth. See (Table 1) for details.

For those with parental data available (*N* = 744), parental gender was 61% female, the largest age group was 40–49 years (64%) followed by 50–59 years (25%). The mean score on PSS parental rewards was 38.5 (standard deviation (SD) 2.3) and 20.9 (SD 6.8) on PSS parental stressors.

### Quality of life

During the 7–9th week of lockdown (T1), youth reported the following mean *T*-scores on KIDSCREEN-27 subscales: physical wellbeing 42.4 (SD 9.2); psychological wellbeing 42.4 (SD 8.6); autonomy and parent relation 50.5 (SD 9.0); social support and peers 44.6 (SD 9.2); school environment 41.8 (SD 9.0). Table 1 shows the subscale scores stratified by gender. For all subscales, girls reported lower scores than boys (all *p*-values < 0.001). These *T*-scores are comparably lower on all subscales than Swedish pre-pandemic norms, and also lower than pre-pandemic European norms except for autonomy and parent relations. Results are shown in Supplementary Information Table 2.

### Change in quality of life over time

Table 2 shows the change in subscale *T*-scores from T1 to T2. The subscale physical and psychological wellbeing subscale scores declined significantly from March to December 2020. Contrary, the subscale score for social support and peers increased in the same period. Neither separate adjustments for age, gender, country of birth, living arrangement, parent laid off, worries about oneself or family getting infected, and worry about the future nor adjustment for all covariates changed the estimates.

### Interaction between time and covariates on levels of quality of life

Significant interaction effects were found for the subscales of physical wellbeing, autonomy, and parent relation and school environment (Table 3).

### Physical wellbeing

**Fig. 1:**
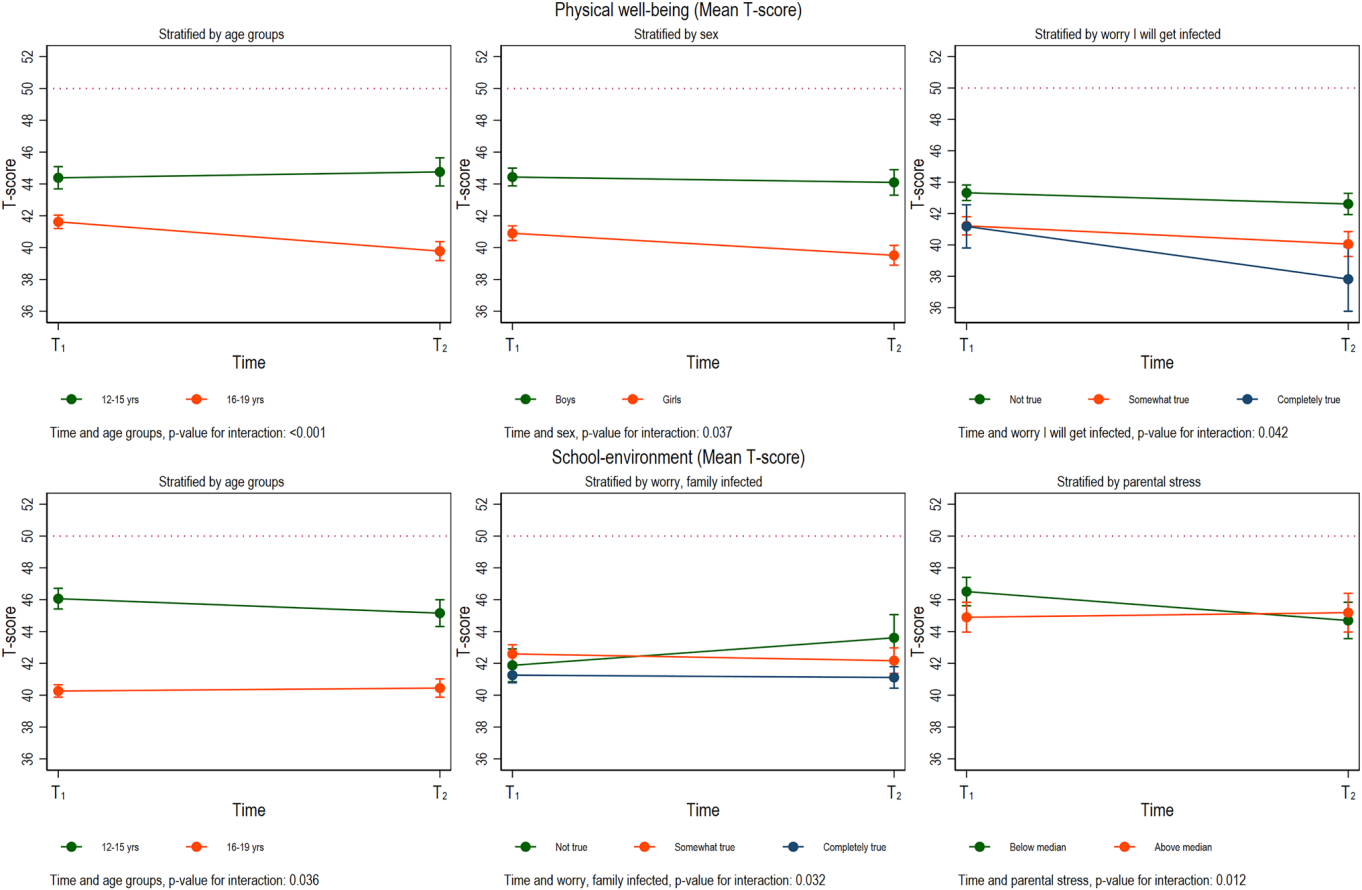
Factors ‘physical wellbeing’ and ‘school-environment’ stratified by covariates with a significant interaction with time. Results from linear mixed models. *T*-score of 50 indicated by a red dotted horizontal line

As shown in (Table 4 and Fig. 1), scores in physical wellbeing declined more over time for those reporting worry about infection (somewhat/completely true) than those not worried. Those not worried had a higher initial score than those who reported worries. Further, the older age group and girls reported lower initial scores and had a steeper decline than the younger age group and boys.

### Autonomy and parent relation

**Fig. 2:**
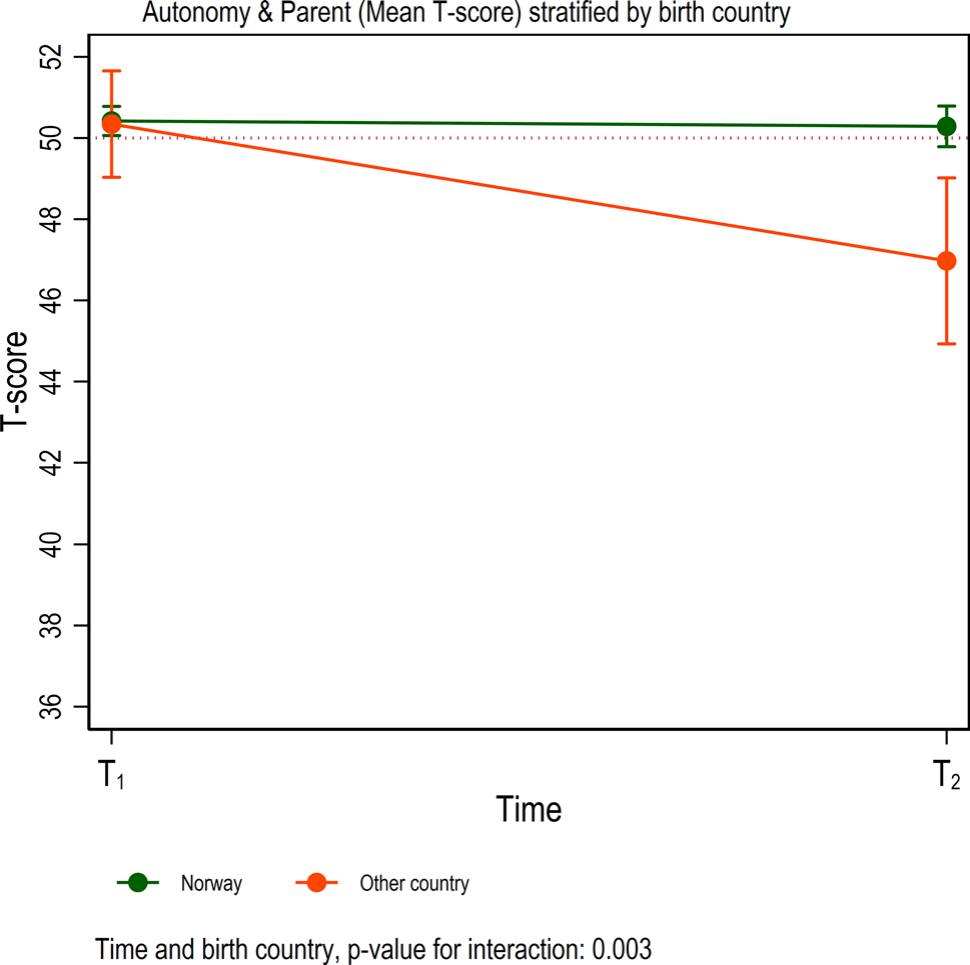
Factor ‘autonomy and parent’ stratified by the birth of the country. Result from linear mixed models. *T*-score of 50 indicated by a red dotted horizontal line

Table 4 and Figure 2 show that even though youth born outside Norway had similar scores on autonomy and parent relation to those born in Norway at T1, their score declined significantly more by T2.

### School-related QoL

The older age group showed a significantly steeper decline in the school environment subscale than the younger group. In addition, among the youngest cohort aged 11–13 years, parental stressors below median yielded a steeper decline in school environment scores from T1 to T2, compared to youth where parents reported stressors above the median.

## Discussion

By examining multiple dimensions of HRQoL among young people, this longitudinal study extends and nuances the emerging knowledge on HRQoL during the COVID-19 pandemic. Compared to pre-pandemic European norms of the KIDSCREEN-27 [11], the youth in this study had lower scores on all dimensions in the first weeks of the national lockdown, except for the autonomy and parent relation dimension. Scores on physical and psychological wellbeing declined further during the following 9-month period of the pandemic. However, the peers and social support scores increased in the same period. Controlling for several sociodemographic- and COVID-19-related factors did not change the overall trends. The results add to several previous European studies that have mostly reported a reduction in HRQoL in children and adolescents during the pandemic [17, 18], suggesting differential effects of the COVID-19 pandemic depending on the dimension of wellbeing in question. In contrast, a German study found no clear change [20].

Our finding that youth in the first weeks of the pandemic had a lower HRQoL score than both Swedish and European pre-pandemic norms on most of the dimensions [11], is in line with other studies conducted in Norway during the first weeks of the lockdown [19, 20]. However, the current study complements these findings by documenting a decline in physical and psychological wellbeing from March to December 2020. Moreover, our findings of a decline in psychological wellbeing mirrors earlier reported results from the same study sample showing increased internalizing symptoms over time [6]. These results draw a worrying picture of potential adverse effects on the mental health of the COVID-19 pandemic for youth in Norway.

A more pronounced decrease in physical wellbeing was observed for those reporting worries about the infection compared to those not worried. The most worried participants also had lower physical wellbeing scores initially. One can only speculate, but it might be that youth reporting worries about infection, are also those with genuine concerns regarding their somatic health, thereby reporting relatively lower physical wellbeing. Due to the risk of detrimental consequences of infection with COVID-19 for people with existing co-morbidity, these youth might experience “double jeopardy”: By being confined to inactivity and isolation to a larger degree than most youth during the outbreak, they may be more prone to deteriorating physical wellbeing throughout the pandemic.

In addition, girls reported lower initial physical wellbeing, with a steeper decline over time compared to boys. These findings are partly in line with pre-COVID-19 findings, which have shown that boys score higher on physical wellbeing [35]. As indicated by our findings, a steeper decline for girls during the COVID-19 pandemic is a matter of concern, as they initially report lower levels of physical wellbeing. Only a few studies have examined sex differences in HRQoL during COVID-19 [19], and sex differences in physical wellbeing have not been examined. However, falling levels of physical activity and increased time spent on sedentary pursuits after the COVID-19 outbreak, with typically lower levels of physical activity among girls have been reported [36, 37]. Our findings thus add to the concern of negative health effects also on physical facets related to the COVID-19 outbreak. This should be of utmost interest for future studies, with special attention to the situation of girls.

The oldest age group also showed a significantly steeper decline in school-related QoL than the younger cohort. This is in line with our previous finding from the first wave, where the older youth reported being more impacted by schools getting closed and learning less [8]. Collectively, these results may indicate that high-school students were particularly vulnerable to the consequences of lockdown and online schooling. This group is at an age where school grades are essential for further academic opportunities. Simultaneously, parental academic support and guidance might be less available on a higher educational level. Interestingly, those with parents scoring below the median on parental stressors had higher scores on school-related wellbeing at T1 relative to youth with parents reporting higher parental stressors. The school environment subscale measures youth’s perception of cognitive capacity, learning and concentration, and positive feelings about school and relations with teachers. Thus, more stressed parents seem to be detrimental to these experiences. However, youth with parents reporting low parental stressors had a steeper decline in school-related QoL, resulting in more similar scores to peers with more stressed parents. It might seem that the potential protective effect of low parental stressors in the initial phase of the pandemic did not last over an extended period.

Nevertheless, social relations with friends and peers and perceived social support increased during this period regardless of age. One may speculate that the ease of national and local preventive measures during fall 2020 may have positively influenced HRQoL in these domains. Even if young people still experienced uncertainty regarding physical school attendance and access to leisure activities, they had more face-to-face social contact compared to the period of the national lockdown in March. T2 data collection was completed between the 16th of December 2020 and the 10th of January 2021, thus including the Christmas season. It is possible that youth were more engaged in joyful activities and that this could have influenced HRQoL. On the other hand, the pandemic Christmas season might have been difficult for many young people, because social activities and family gatherings were strictly limited. We do not know if and to which degree this impacted HRQoL among young people.

The parent relations and autonomy dimension scores were comparable to European pre-pandemic norms. The relatively high scores imply that the youths generally experienced supportive relationships with their parents during the pandemic. This is an important finding because a positive atmosphere at home and feelings of having enough age-appropriate freedom are essential for healthy youth development and are protective factors during stress and uncertainty [38]. Still, a positive relationship with parents did not prevent an average drop in psychological and physical wellbeing over time. Noticeably, the parent relations and autonomy dimension declined significantly from March to December for youth born abroad. This negative change might reflect that immigrant parents have been generally more negatively affected by the pandemic than the non-immigrant population. Immigrants are over-represented in the sectors of the labor market that were most vulnerable to lay-offs. Further, immigrant families more often live in low-income households. They might have had fewer resources than native-born parents to cope with home-schooling, including computer- and internet access and knowledge. Language difficulties might also have made home-schooling difficult for many immigrant families. This interpretation concurs with other research documenting that children and youth from families with low socioeconomic status, immigrant background, and limited living space were more affected by COVID-19 regarding decreases in health-related quality of life [17, 39]. One could assume that several of these aspects may impact the current study’s parent relations and autonomy dimension.

### Strengths and limitations

A strength of this study is the large sample of respondents and its longitudinal design during the pandemic. The KIDSCREEN-27 is a validated measure of HRQoL for adolescents and has shown acceptable test–retest reliability, criterion, and construct validity [12], also enabling comparison to European norm data [11]. However, since the present study does not include a pre-pandemic assessment to enable non-pandemic comparison conditions, it is not possible to purely ascribe the observed changes to the impact of COVID-19. Also, despite a large sample, the response rate of 40% with high attrition at T2 puts limitations on the generalizability of our findings and may have biased our results. Further, the data were self-reported and thus prone to recall bias and social desirability.

We did not have the opportunity to match parent-completed PSS to youth self-reported HRQoL for cohort 2, aged 16–19 years. Therefore, our findings of a steeper decline in school environment scores among youth where parents reported low parental stressors may not be fully generalizable to older youths. Also, the youngest cohort had parents with higher income and educational level than the non-consenting parents. The findings should therefore be generalized with caution.

We do not have information on the participants somatic health. Such data could have contributed to explain the association between low physical HRQoL scores and pandemic worries.

Finally, we cannot exclude the possibility of some degree of bias in the presented regression estimates because of the missing data rates for some of the included variables in this paper. Given the number of interactions, we investigated, generating multiple imputation models accounting for the interactions and the variables of interest was deemed intractable.

## Conclusions

This study contributes to understanding how the COVID-19 pandemic has influenced youths’ quality of life in different domains. Most noticeable, this study adds to previous studies on HRQoL during the COVID-19 pandemic by demonstrating differential findings for the various wellbeing dimensions among young people in Norway. One concern is that physical and psychological wellbeing seemed impaired early in the lockdown and declined further during the following 9 months, possibly interfering with normal developments. On the other hand, for the overall sample, the autonomy and parent relation dimension seemed not to be affected by the pandemic in the current study. The scores on the peers and social support dimension increased during this period. School personnel, trainers, and other adults in contact with youth are gatekeepers for youth to access relevant social and mental health services. Steps should be taken to enable adults working with young people to identify those not recovering swiftly from the impact of the pandemic as society goes back to a more normal state. Particular attention is warranted for girls, immigrants, and older youths.

## Supplementary Information

Below is the link to the electronic supplementary material.

Supplementary file1 (DOCX 17 KB)

Supplementary file2 (DOCX 16 KB)
