# Supplementary material for: Quality of life among young people in Norway during the COVID-19 pandemic. A longitudinal study
Source: Eur Child Adolesc Psychiatry. 2022 Jun 22;32(6):1061–71. doi: 10.1007/s00787-022-02023-5 (PMC9216295; doi:10.1007/s00787-022-02023-5)
Supplement: Supplementary file 1 — Supplementary file1 (DOCX 17 KB) [file 787_2022_2023_MOESM1_ESM.docx]

**Supplementary information**

Article title: Quality of life among young people in Norway during the COVID-19 pandemic. A longitudinal study.

Journal name: European Child and Adolescent Psychiatry

Author names: Stine Lehmann, Ellen Haug, Ragnhild Bjørknes, Gro Mjeldheim Sandal, Lars T. Fadnes, Jens Christoffer Skogen

Corresponding Author: Stine Lehmann, Department of health promotion and development, Faculty of psychology, University of Bergen, Postboks 7807, 5020 Bergen, Norway. E-mail: [stine.lehmann@uib.no](mailto:stine.lehmann@uib.no)

Table2: T-scores on sub-scales of Health related Quality of life (HRQoL) in study sample compared to other youth groups using the KIDSCREEN-27.

|  | Study data | | | Swedish data | | | European norms | | | Study data  VS  Swedish | Study data  VS  European norms |
| --- | --- | --- | --- | --- | --- | --- | --- | --- | --- | --- | --- |
|  | N | M | SD | N | M | SD | N | M | SD | Cohen`s d | Cohen`s d |
| Physical well-being^1^ | 2584 | 42.4 | 9.2 | 202 | 48.8 | 9.2 | 15239 | 48.6 | 9.6 | **-0.70 (-0.84,-0.55)** | **-0.65 (-0.69,-0.60)** |
| Psychological well-being^1^ | 2574 | 42.6 | 8.6 | 202 | 53.4 | 10.9 | 15323 | 48.8 | 9.8 | **-1.23 (-1.38,-1.09)** | **-0.65 (-0.69,-0.60)** |
| Autonomy & Parent relation^1^ | 2550 | 50.5 | 9.0 | 202 | 55.1 | 9.9 | 15135 | 49.4 | 9.8 | **-0.51 (-0.66,-0.37)** | **0.11 (0.07,0.15)** |
| Social support & Peers^1^ | 2560 | 44.6 | 9.2 | 202 | 54.1 | 8.2 | 15372 | 49.6 | 10.0 | **-1.04 (-1.19,-0.90)** | **-0.51 (-0.55,-0.47)** |
| School-related^1^ | 2555 | 41.8 | 9.0 | 202 | 55.8 | 9.6 | 15255 | 48.4 | 9.4 | **-1.55 (-1.70,-1.40)** | **-0.71 (-0.75,-0.67)** |

^1^ T-score at baseline

Bold indicates statistical significance at p<0.001.
