# Supplementary material for: Quality of life among young people in Norway during the COVID-19 pandemic. A longitudinal study
Source: Eur Child Adolesc Psychiatry. 2022 Jun 22;32(6):1061–71. doi: 10.1007/s00787-022-02023-5 (PMC9216295; doi:10.1007/s00787-022-02023-5)
Supplement: Supplementary file 2 — Supplementary file2 (DOCX 16 KB) [file 787_2022_2023_MOESM2_ESM.docx]

Supplementary information

Article title: Quality of life among young people in Norway during the COVID-19 pandemic. A longitudinal study.

Journal name: European Child and Adolescent Psychiatry

Author names: Stine Lehmann, Ellen Haug, Ragnhild Bjørknes, Gro Mjeldheim Sandal, Lars T. Fadnes, Jens Christoffer Skogen

Corresponding Author: Stine Lehmann, Department of health promotion and development, Faculty of psychology, University of Bergen, Postboks 7807, 5020 Bergen, Norway. E-mail: stine.lehmann@uib.no

Table 1: Comparison between those participating only at baseline versus those participating on both time points.

| **Characteristic** | **Only baseline^1^** | **Both timepoints^1^** | **p-value^2^** |
| --- | --- | --- | --- |
| Gender |  |  | 0.026 |
| Boy | 504 (43%) | 581 (39%) |  |
| Girl | 663 (57%) | 912 (61%) |  |
| Age groups |  |  | <0.001 |
| 12-15 yrs | 250 (21%) | 451 (30%) |  |
| 16-19 yrs | 917 (79%) | 1,042 (70%) |  |
| Birth country |  |  | 0.094 |
| Norway | 1,074 (92%) | 1,399 (94%) |  |
| Other country | 93 (8.0%) | 94 (6.3%) |  |
| Living arrangements |  |  | 0.002 |
| Both parents | 925 (79%) | 1,257 (84%) |  |
| Mother or father | 214 (18%) | 199 (13%) |  |
| Other | 28 (2.4%) | 37 (2.5%) |  |
| Parent laid off (% yes) | 224 (25%) | 190 (19%) | <0.001 |
| Worry, family infected |  |  | 0.6 |
| Not true | 130 (11%) | 166 (11%) |  |
| Somewhat true | 405 (35%) | 541 (37%) |  |
| Completely true | 632 (54%) | 773 (52%) |  |
| Worry I will get infected |  |  | 0.3 |
| Not true | 626 (54%) | 802 (54%) |  |
| Somewhat true | 450 (39%) | 585 (40%) |  |
| Completely true | 91 (7.8%) | 92 (6.2%) |  |
| Worry about future |  |  | 0.7 |
| Not true | 538 (46%) | 694 (47%) |  |
| Somewhat true | 397 (34%) | 508 (34%) |  |
| Completely true | 232 (20%) | 275 (19%) |  |
| Physical well-being | 42 (9) | 43 (9) | 0.2 |
| Psychological well-being | 43 (9) | 43 (9) | 0.9 |
| Autonomy & Parent relation | 50 (9) | 51 (9) | 0.6 |
| Social support & Peers | 45 (9) | 44 (9) | 0.2 |
| School-related | 41 (9) | 42 (9) | 0.001 |
| *^1^* n (%); Mean (SD)  *^2^* Pearson's Chi-squared test; Welch Two Sample t-test | | | |
